# Evaluation of Regeneration Potential of Bone Marrow–Derived Mesenchymal Stem Cells on Induced Damaged Submandibular Salivary Gland in Mice

**DOI:** 10.1055/s-0044-1791940

**Published:** 2025-03-12

**Authors:** Nadia Attia Radi, Doaa Adel Habba, Seham Ibrahim Hallool, Ahmed Ali Almeshari, Hanaa Mohamed Abd Elsamia

**Affiliations:** 1 Department of Oral and Dental Pathology, Faculty of Dental Medicine for Girls, Al Azhar University, Cairo, Egypt; 2 Department of Oral and Maxillofacial Surgery and Diagnostic Sciences, College of Dentistry, Najran University, Kingdom of Saudi Arabia; 3 Department of Oral and Dental Biology, Faculty of Dental Medicine for Girls, Al Azhar University, Cairo, Egypt; 4 Department of Oral and Maxillofacial Pathology, Faculty of Oral and Dental Surgery and Medicine, Zagazing University, Zagazing, Egypt

**Keywords:** BM-MSCs, Ki-67, CD34

## Abstract

**Objectives** The ultimate goal of stem cell (SC) transplantation is the regeneration of salivary gland function by transplanted SCs differentiating into salivary gland cells. Therefore, this study aimed to evaluate the regenerative capacity of bone marrow–derived mesenchymal stem cells (BM-MSCs) transplantation in irradiated mice using the immunohistochemical markers Ki-67 and CD34.

**Material and Methods** Four groups of male mice were included in the study. Group I (normal control) comprised six mice that were not subjected to gamma radiation. Group II comprised six irradiated mice that were not treated with BM-MSCs. Group III comprised 12 irradiated mice that were treated with intraglandular injection of labeled BM-MSCs into their submandibular salivary glands, 24 hours postradiation. Group IV comprised 12 irradiated mice that were treated with intraglandular injection of labeled BM-MSCs into their submandibular salivary glands, on day 11 postradiation.

**Statistical Analysis** Data were presented as mean and standard deviation. The different groups were compared using a one-way analysis of variance (ANOVA).

**Results** The ANOVA test revealed that the difference between all groups was extremely statistically significant ( *p* < 0.003), and Tukey's post hoc test revealed a statistically significant difference between group II and groups I, III, and IV included in the study regarding microvessel density of CD34 immunoexpression in different groups.

**Conclusion** BM-MSCs have a regeneration potential on induced damaged submandibular salivary glands in mice; time is an essential factor in the regeneration capacity of BM-MSCs.

## Introduction

The salivary gland (SG) is made up of a complex network of ductal systems and secretory units that work together to generate and transport saliva, which plays an essential role in preserving oral and general health. 1 2 Numerous conditions can impair the SG's ability to function normally, including radiation damage, autoimmune diseases, certain systemic conditions like diabetes and neurological diseases, and a wide range of medications like antihypertensives, β-blockers, and antidepressants. 3 4 Ionizing radiation (IR) induced SG damage includes SG duct dilation, atrophy of the acinar cells, a reduced number of secretory granules, and contraction of the SGs. The damage caused to the SG by IR exposure is a dynamic cascade that occurs in two major phases of response to damage: the initial acute phase and the long-term chronic phase. There are two primary regenerative techniques that have been investigated to restore the function of the SG that has been damaged by radiation. The first technique involves applying tissue engineering concepts to create an artificial SG. A second strategy has attempted to treat damaged SG tissue using stem cell–based therapy. The utilization of stem cells' special abilities, such as differentiation and self-renewal, in therapy is a novel technique to repair or replace damaged human tissues and cells with new, fully functional, and healthy ones. 5 6 Bone marrow–derived mesenchymal stem cells (BM-MSCs) are multipotent stem cells that are thought to be the ideal source for organ and tissue repair. BM-MSCs may display several therapeutic functions to support the regeneration and repair of damaged tissues, including the following:

- *Direct differentiation* : They can migrate to the site of injury, where they can differentiate and replace the injured resident cells and facilitate tissue regeneration.
- *Paracrine effects* : These cells have the ability to secrete soluble substances involving growth factors, cytokines, chemokines, regulatory factors, and nucleic acids, which are essential for the survival and division of cells and for modulating the immune response.

It has been shown that direct intraglandular transplantation of BM-MSCs after radiation can repair the injured gland and enable it to function again by differentiating into cells that produce saliva and secrete paracrine substances. 7 8 9 The nuclear antigen Ki-67 is a highly reliable indicator of active cell proliferation in both tumor and normal cell populations. It is thought to be the most appropriate biological marker of mitotic activity due to its nuclear expression at a specific cell cycle phase. Evaluation of proliferation markers is quite valuable in pathological diagnosis and prognosis. It has been shown that Ki-67 has a prognostic character for many types of malignant tumors, such as colorectal, prostate, and breast cancers. 10 11 CD34 is a transmembrane phosphoglycoprotein that is found on the cell surface in humans and other animal species. Moreover, it has been found that CD34 proteins have a role in inhibiting progenitor cell differentiation, which is essential for preserving a reserve of immature cells that can differentiate into other cell types as needed. Certain studies have suggested that CD34 may act as a positive biomarker for MSCs, with a particular connection to vascularization. These cells can be referred to as vascular progenitor cells, implying that they can differentiate into endothelial cells and aid in the formation of blood vessels. 12 13 The ultimate goal of stem cell transplantation is the regeneration of SG function by transplanted stem cells differentiating into SG cells. So, this study aimed to evaluate the regenerative capacity of BM-MSC transplantation in irradiated mice using the immunohistochemical markers Ki-67 and CD34.

## Materials and Methods

### Animals

Forty-six adult male mice weighing between 18 and 25 g and aged between 8 and 12 weeks were used in this study. Everything from the facilities to the food to the scarification method was done in compliance with the guidelines set forth by the ethical committee for animal experimentation. The Ethics Committee of the Faculty of Dental Medicine at Al-Azhar University provided the study's ethical code and approval (code no. REC-PD-PD-24–06).

### Experimental Groups

- **Group I** (normal control) included six mice that were not subjected to gamma radiation.
- **Group II** (positive control) included six irradiated mice that were not treated with BM-MSCs.
- **Group III** included 12 irradiated mice that were given an intraglandular injection of labeled BM-MSCs 24 hours after the radiation treatment.
- **Group IV** included 12 irradiated mice given an intraglandular injection of labeled BM-MSCs on day 11 after radiation therapy for their submandibular SGs.

### Isolation and Expansion of Mice BM-MSCs

Ten mice were used; their tibias and femurs were used to obtain bone marrow samples, from which BM-MSCs were isolated and later identified by phenotyping and flow cytometric analysis. The PKH26 Red Fluorescent Cell Linker Kit (Sigma Aldrich, St. Louis, MO, United States) was then used to label BM-MSCs. Isolation and expansion of mice BM-MSCs were performed in the Biochemistry Department, Faculty of Medicine, Cairo University, Egypt. The procedures for isolation of BM-MSCs follow the protocol described by Snykers et al 14 and Dvorakova et al. 15

### Irradiation Process

The irradiation process was performed at the National Center for Radiation Research and Technology (NCRRT), Atomic Energy Authority, Cairo, Egypt, using an Indian Gamma Cell (Ge 4000 A) giving a dose rate of 1.538 kGy/h. The adult male mice were anesthetized generally with thiopental sodium (Egyptian Pharmaceutical International Company [EPICO], which was purchased from a local market), ultra-short-acting barbiturate (60 mg/kg) injected intraperitoneally (i.p.). Then, the mice were locally irradiated with a single dose of 15 Gy in the region of the head and neck, with the rest of the body shielded with lead. 16 17 18 The irradiated mice were randomly divided into groups II, III, and IV.

### Intraglandular Transplantation of BM-MSCs

Both groups II and III were usually given an intraperitoneal injection of thiopental sodium (60 mg/kg) to induce anesthesia 24 hours after radiation. A vertical incision was made in the neck to expose the submandibular SGs, and then the skin was gently retracted. Subsequently, each submandibular gland (SMG) of group III mice was directly injected with 1 × 105 PKH26-labeled BM-MSCs in 50 µL of phosphate-buffered saline (PBS), while group II mice's SMGs were just treated with 50 µ of PBS. The surgical wound was sutured using an absorbable synthetic surgical suture after the injection-coated VICRYL braided suture (5–0).

Penicillin G powder was then applied to seal the incision. On day 11 postradiation, the mice belonging to group IV were put under anesthesia and injected with BM-MSCs using the previously described method.

### Specimen Collection

On day 30 postradiation, six animals from each treated group (III and IV) were anesthetized, and their glands were examined using florescent microscopy to trace the PKH26-labeled cells (red fluorescent dye) to confirm their presence in the glands. On day 90 (D90), following radiation, the mice from each group were anesthetized and subsequently sacrificed individually through cervical dislocation. After that, the SMGs were collected and preserved in 10% formalin buffer. One gland was prepared and histologically examined for each sacrificed animal, and the other gland was ready for immunohistochemical labeling with Ki-67 and CD34 antibodies.

### Specimen Preparation

**For histological examination** , the specimens underwent regular dehydration, clearing, and embedding in paraffin wax. After that, sections of 4-μm thickness were cut, stained with hematoxylin and eosin (H&E) stain, and inspected under a light microscope with a magnification of ×200.

**For immunohistochemical staining** , paraffin sections of 4-μm thickness were cut and mounted on adhesive glass. Sections were deparaffinized with xylene, rehydrated, and washed with PBS. Sections were incubated in 0.3% H _2_ O _2_ for 10 minutes to block the activity of endogenous peroxidase. After immersing the slides in a citrate buffer solution with pH of approximately 6, they were subjected to 5-minute microwave bursts at 95°C three times before being cleaned with PBS to expose antigen. Sections were then treated with the indicated primary antibodies: Ki-67 mouse monoclonal antibody (Clone: PP-67: P6834 in a dilution range of 1:800 and was manufactured by Sigma Aldrich) and CD34, a mouse monoclonal antibody (clone ICO115: sc-7324) in a dilution of 1:50 (Dako, Life Trade, Egypt). After this, the sections were washed several times with PBS. The sections were incubated with secondary biotin-coupled antibodies for 5 to 10 minutes at room temperature, washed several times with PBS, and immunolabeled with diaminobenzidine (DAB). 19 The tissues were inspected using light microscopy at ×200 magnification.

### Histomorphometric Analysis

The immune-stained sections were examined using a light microscope to assess the prevalence of positive cases and the location of immunostaining within the tissue. The stained cells were considered to be Ki-67-positive if there was cytoplasmic and/or nuclear staining. Computerized image analysis using a Leica image analyzer (Germany) was used. The immunoreactivity of Ki-67 was determined by estimating the proportion of immunostained cells in relation to the area. The image analyzer was automatically adjusted to transform the measurement units (pixels) generated by the image analyzer program into real micrometer units. The percentage of positive immune reactive area of Ki-67 was calculated in relation to a reference measuring frame of area 11,434.9 μm ^2^ using magnification (×200). Five fields were obtained sequentially from each slide for histomorphometric evaluation. Then, the mean values for each specimen were determined. Micro-vessel density (MVD) was assessed by detecting capillaries positive for the CD34 antibody.

Five fields of the most vascularized areas at ×40 magnifications were selected as hotspots, and the vessels in each field were counted. MVD for each sample was considered as the mean number of vessels in these areas. Single endothelial cells or clusters of these cells, with or without lumen, were considered individual vessels. The intensity of staining was not considered for evaluation in the studied groups.

### Statistical Analysis

Data were presented as mean and standard deviation (SD). Analysis of variance (ANOVA) followed by post hoc test was used to compare between groups. At *p* ≤ 0.05, the significance level was established. IBM SPSS Statistics version 20 for Windows was used for statistical analysis.

## Results

### Immunofluorescence Results

**Fig. 1:**
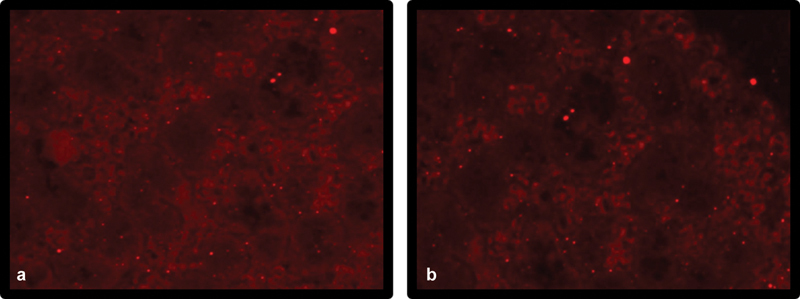
A photomicrograph of the submandibular salivary glands of ( **a** ) group III and ( **b** ) group IV showing survived PKH26-labeled bone marrow–derived mesenchymal stem cells (BM-MSCs) in both groups (immunofluorescence [IF], ×400, red channel).

As shown in Fig. 1 , the immunofluorescent (IF) pictures of the submandibular SGs of groups III and IV on day 30 postradiation show survived PKH26-labeled BM-MSCs in both groups (IF ×400, red channel).

### Histological Results

**Fig. 2:**
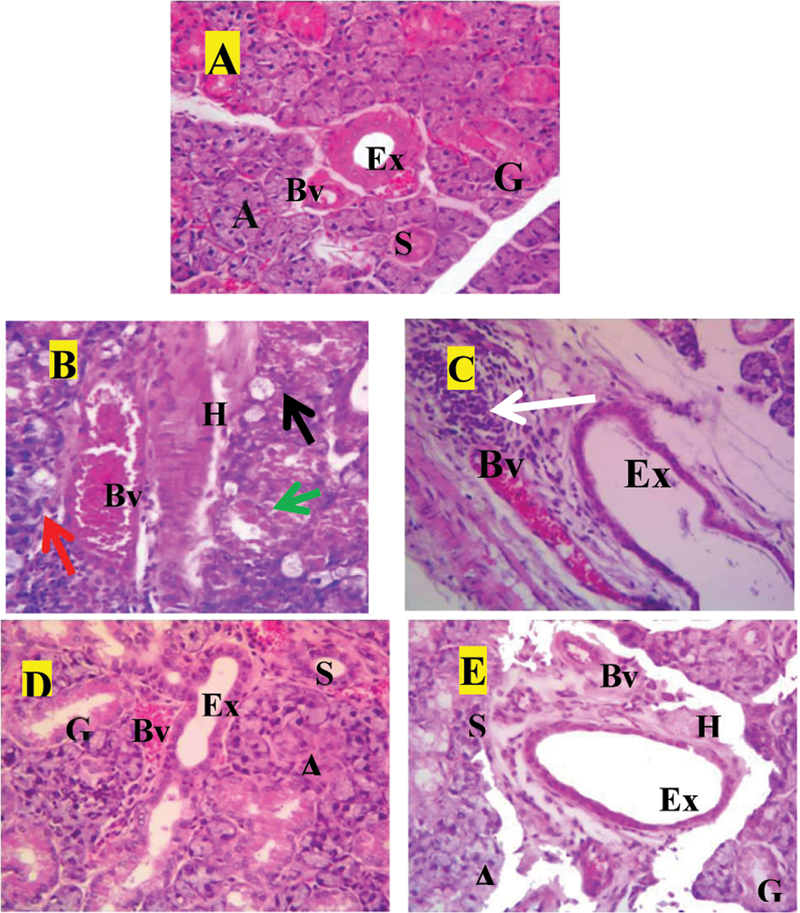
Photomicrographs of the submandibular salivary glands of **(A)** the control group (group I) showing normal gland architecture and structure, striated duct (S), excretory ducts (Ex) lined by pseudostratified columnar epithelium present in fibrous C.T. septa, blood vessel (Bv), acini (A), and granular convoluted tubules (G). ( **B,C** ) Group II showing severely destructed glands with loss of acinar and ductal architecture, vacuolated acinar and ductal cells ( *black arrows* ), cell lysis and cell remnants ( *green arrows* ), large cells with large hyperchromatic nuclei ( *red arrows* ) within the acini, extremely wide interlobular space with excretory duct had dilated lumen and reduction in the height of its lining cells (Ex), severe hyalinization of the supporting stroma (H), inflammatory cells infiltrate ( *white arrows* ), and congested blood vessel (Bv) with abnormally thickened wall and degenerated endothelial lining. ( **D** ) Group III showing greatly more preserved acinar and ductal architecture and cell lining in addition to C.T. stroma compared with group II. ( **E** ) Group IV showing relatively preserved tissue integrity and gland architecture, better than group II but worse than group III (hematoxylin and eosin [H&E], ×400).

As demonstrated in Fig. 2 , examination of H&E-stained sections from the submandibular SGs of the control group (group I) showed normal gland architecture composed of normal serous acini with compact arrangements, granular convoluted tubules (GCTs), and striated ducts associated with few blood capillaries. In addition, the interlobular excretory ducts were detected to be surrounded by connective tissue stroma that contained blood vessels ( Fig. 2A ). While H&E-stained sections from group II at D90 postradiation revealed the glands were considerably damaged, with acinar and ductal architecture lost, acinar and ductal cells vacuolized and lysed, and remnants of cells present. Large cells with notably enlarged, hyperchromatic nuclei could also be observed. In addition, wide interlobular spaces clearly appeared at the expense of acinar shrinkage and atrophy. The excretory ducts showed dilated lumen and reduction in the height of their lining cells, severe hyalinization of the supporting stroma, inflammatory cells infiltrate, and congested blood vessels, with some showing abnormal wall thickening and degenerated endothelial lining ( Fig. 2 B, C ). In group III, submandibular SGs at D90 postradiation had largely normal gland architecture, according to an examination of H&E-stained sections from those glands. The acinar and ductal architecture and cell lining, in addition to connective tissue (CT) stroma appeared greatly more preserved compared with group II. Inflammatory cells infiltrate in the interstitial connective tissue, but markedly less than group II ( Fig. 2D ). Examination of H&E-stained sections from submandibular SGs of group IV at D90 postradiation showed relatively preserved tissue integrity and gland architecture, better than group II but worse than group III. Stromal hyalinization and inflammatory cells infiltrate within the interstitial connective tissue were less than group II but more than group III ( Fig. 2E ).

### Immunohistochemical Results

**Fig. 3:**
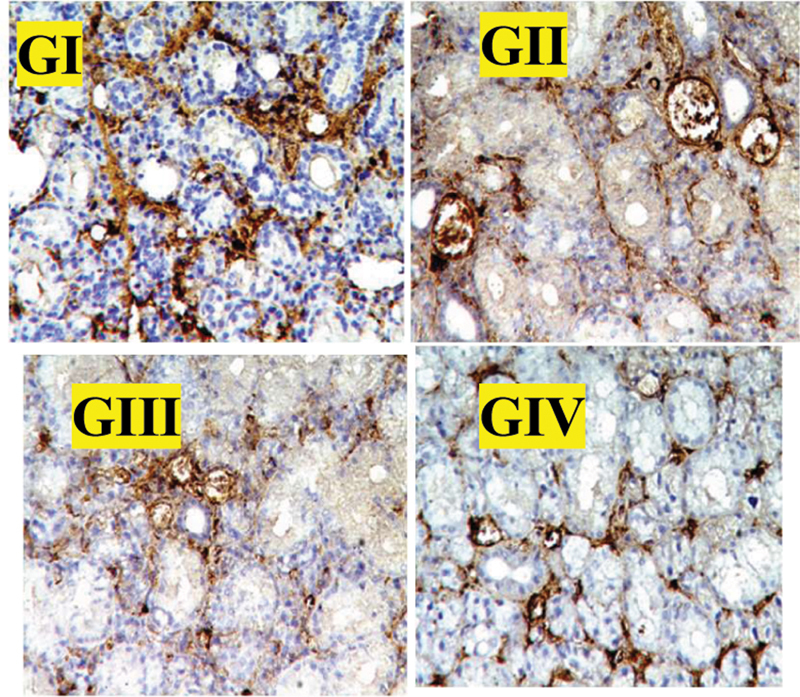
Photomicrographs of CD34 expressions in different groups showing the positive reaction was confined to the endothelial cells lining blood capillaries. The staining positive area for CD34 was significantly higher in group III; this staining positive area was decreased in group IV and group I by comparison with group III, but only weak positive results were obtained in group II (×200).

The positive immune reaction for CD34 was confined to the endothelial cells lining blood capillaries. The staining positive area for CD34 was significantly higher in group III; this staining positive area was decreased in group IV and group I by comparison with group III, but only weak positive results were obtained in group II ( Fig. 3 ; GI–GIV).

**Fig. 4:**
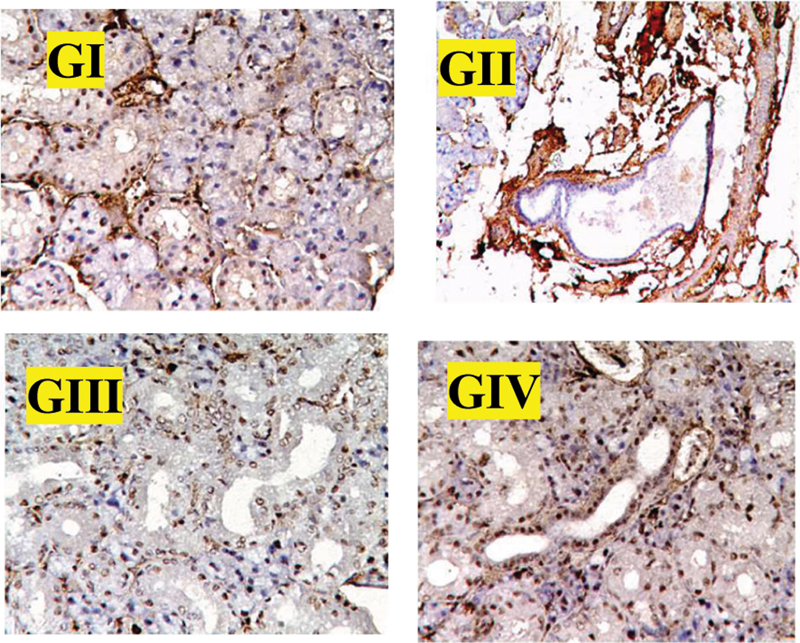
Photomicrographs of Ki-67 expressions in different groups showing the positive reaction for Ki-67 was seen as a brown homogenous stain in the nuclei of acinar and ductal cells, with higher expression in ductal cells. A few positive cells are seen in the unirradiated control group (×200).

A positive immune reaction for Ki-67 was seen as a brown homogenous stain in the nuclei of acinar and ductal cells, with higher expression in ductal cells ( Fig. 4 ; GI–GIV). A few positive cells were seen in the unirradiated control group.

### Statistical Results

**Fig. 5:**
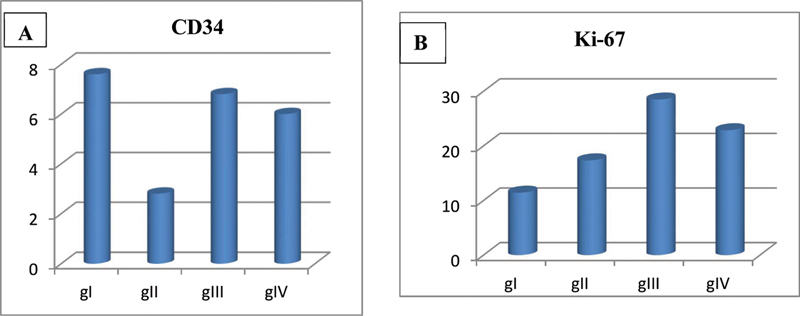
**(A)** Column chart showing mean CD34 microvessel density (MVD) in different groups. **(B)** Column chart showing the mean Ki-67 area percent in different groups.

The ANOVA test revealed that the mean area percentage of MVD of CD34 between all groups was extremely statistically significant ( *p* < 0.003). Group I showed the statistically highest mean MVD, followed by groups III and IV. The lowest mean value was recorded in group II. Tukey's post hoc test revealed a statistically significant difference between group II and groups I, III, and IV included in the study. While the ANOVA test revealed that the difference of Ki-67 mean area percent between all groups was extremely statistically significant ( *p* < 0.000). Turkey's post hoc test revealed statistically significant differences between all groups included in the study ( Table 1 and Fig. 5 ).

**Table 1:** Comparison of Ki-67 area percent between all groups

Group	CD34 Mean ± Std. Dev.	Ki-67 Mean ± Std. Dev.
Group I	7.6000 ^a^ ± 1.94936	11.4220 ^a^ ± 1.77662
Group II	2.8000 ^b^ ± 0.83666	17.3440 ^b^ ± 2.90291
Group III	6.8000 ^a^ ± 2.28035	28.4900 ^c^ ± 2.64593
Group IV	6.0000 ^a^ ± 1.58114	22.8600 ^d^ ± 2.47280

At P ≤ 0.05, the significance level was established. Considering CD34, there was statistical significant difference between gI and gII as *P* value recorded.001 so the two groups had different superscript letters (a,b), while there were no statistical significant difference between gI, III, IV as the *P* values recorded (.479, .167) so the three groups had the same superscript letters (a). There was statistical significant difference between g II and both of gIII and g IV as *P* value recorded (.002, .011) respectively, so these groups had different superscript letters (b,a) respectively. Considering Ki-67, there were statistical significant difference between all groups included in the study as the *P* value recorded were less than 0.05,so the groups has different superscript letters (a,b,c,d).

Abbreviations: ANOVA, Analysis of Variance, ANOVA tests indicated extremely statistically significant differences for both Ki-67 (p < 0.000) and CD34 MVD (p < 0.003) between all groups. MVD, Micro-vessel density; SD, Standard deviation.

Note: Analysis of variance (ANOVA) test revealed that the difference between all groups was extremely statistically significant ( *p* < 0.000). Tukey’s post hoc test revealed statistically significant differences between all groups.

*Significance at *p* < 0.05. Tukey's post hoc test: means with different superscript letter are statistically significantly different.

Comparison of CD34 MVD between all groups. ANOVA test revealed that the difference between all groups was extremely statistically significant ( *p* < 0.003). Group I showed the statistically highest mean MVD followed by groups III and IV. The lowest mean value was recorded in group II. Tukey's post hoc test revealed a statistically significant difference between group II and groups I, III, and IV.

## Discussion

Among the most dangerous side effects of radiation treatment is damage to the SGs, which can lead to severe hypofunction and irreversible xerostomia. As such, several regenerative treatment approaches, including stem cell–based therapy, growth factors, tissue engineering, and gene therapy, have been tested. 20 In the present research, acinar and ductal cell lysis, loss of acinar and ductal architecture, vacuolization, and the presence of cell remnants were found in severe submandibular SG damage of group II on D90 following radiation exposure, as shown by light microscopic analysis of H&E-stained sections from these glands. Furthermore, there was a noticeable appearance of wide interlobular space at the expense of acinar atrophy. All these histologically obtained results were in accordance with other previous studies. 21 22 Radiation-induced cellular deoxyribonucleic acid (DNA) impairment and reactive oxygen species (ROS) production may be the cause of apoptosis, which can result in acinar cell lysis and death. Furthermore, the deteriorating changes observed at D90 (late phase) postirradiation may be the consequence of a gradual loss of cells, which is linked to a lack of their replenishment as a result of radiotherapy-induced damage to a population of SG stem/progenitor cells, resulting in the disruption of normal tissue balance. 23 24 Furthermore, the emergence of large cells with large, hyperchromatic nuclei inside the acini was another noteworthy observation made in group II at D90 postradiation in the current study. This discovery aligned with earlier research that, at 8 weeks postradiation, large cells with noticeably enlarged, hyperchromatic nuclei morphologically resembling cells going through senescence started to appear. These cells' appearance increased gradually and became more noticeable at 12 weeks (90 days) postradiation. According to their findings, radiation exposure to the SG in both humans and mice results in cellular senescence, which is an innate and chronic reaction. Additionally, the lining cells in the excretory ducts had a decrease in height and a widening of their lumen. This finding was in agreement with the previous report, which stated that as a result of IR, the duct of the SG becomes dilated and undergoes squamous metaplasia of its lining. 25 26 Furthermore, the connective tissue stroma showed hyalinized collagen bundles adjacent to dilated, congested blood vessels, with some blood vessels showing thickening in their walls. These results were consistent with earlier research that found that extracellular matrix components have been shown to thicken in response to IR. 27 28 By limiting the flow of nutrients, essential minerals, and oxygen to parenchymal cells, these stromal changes may impair aerobic respiration and cause cell death. Moreover, this might negatively impact the functioning of surviving acinar cells' later attempts at regeneration. Moreover, degenerated endothelial cells were also noticed. This was coincident with another study that reported that radiation leads to ROS generation that causes damage to endothelial cells via superoxide. The presence of inflammatory cells infiltrate within the connective tissue stroma in the current study was in line with prior research, which reported the presence of an inflammatory reaction in the early phase postradiation, starting within hours of radiation and continuing later on (during intermediate and late D90 phases) as chronic inflammation that persists. 29 30 On the other hand, in group III, the gland architecture appeared to be largely normal, with a slight infiltration of inflammatory cells in the CT stroma and significantly better preserved than in group II. These results were in line with earlier research that found that intracellular transplantation of BM-MSCs 24 hours after radiation therapy could prevent apoptosis and promote the growth of a range of cells, including endothelial and salivary stem/progenitor cells via paracrine actions, as well as cells with the capacity to transdifferentiate into salivary epithelial cells. The secretion of multiple soluble factors has been reported as the mechanism by which BM-MSCs exert their paracrine actions. Among these are interleukin-6, which improves the repair of DNA damage, and angiopoietin-1, which increases the permeability of epithelial cells. It is possible to interpret the clear decrease in the infiltrate of inflammatory cells as a result of the potent anti-inflammatory soluble factors secreted by BM-MSCs. 9 31 32 Considering the group IV results in the current study, we showed comparatively preserved tissue integrity and gland architecture, better than group II but worse than group III. Stromal hyalinization and inflammatory cell infiltration within the interstitial connective tissue were less than those in group II but more than those in group III. According to these results, the transplantation of BM-MSCs that took place 24 hours after radiation was more helpful for tissue regeneration almost from the start of the early radiation damage phase than transplantation on the 11th day after radiation. This could be explained by the fact that the transplanted BM-MSCs were able to reduce the acute inflammatory response that follows radiation 24 hours later, owing to their potent anti-inflammatory and immunomodulatory properties. 33 34 The difference in Ki-67 mean area percent between all groups was found to be highly statistically significant in this study, as demonstrated by the immunohistochemical examination using staining of Ki-67 antibodies. The highest value was found in group III, followed by groups IV and II, and the lowest value was found in the control group. This finding aligned with an earlier investigation that demonstrated a statistically significant increase in the mean percentage expression of Ki-67 in the MSC-treated group relative to the positive control group. 35 Furthermore, a highly statistically significant difference was found between all groups based on the immunohistochemical analysis of CD34 immunoexpression in each group. Group I exhibited the statistically significant highest mean MVD, following groups III and IV, while group II had the lowest mean value. These were in agreement with previous studies that reported that radiation leads to ROS generation that causes damage to endothelial cells (apoptosis and loss) via superoxide (O _2_^-^ ). O _2_^-^ causes inactivation of NO, which possesses vasoprotective actions, by the formation of peroxynitrite (ONOO ^-^ ), which is a potent oxidant that damages proteins, lipids, and DNA irreversibly. This leads to the disruption of important cell signaling pathways and promotes endothelial cell death. This damage is manifested by microvascular dysfunction, increased capillary permeability, local inflammation, and a decrease in MVD. 36 37 38 It has been previously demonstrated that a single 15-Gy radiation dose results in a 60% reduction in salivary flow rate, primarily due to changes in microvascular endothelial cells within the SGs. This effect is mediated by ceramide and ROS generation, leading to impaired SG function. BM-MSCs can improve the regeneration process of damaged SGs by enhancing the α-amylase activity, which serves as a marker for tissue regeneration in the SGs. Additionally, angiopoietin-1, which is secreted by BM-MSCs, has been shown to facilitate the recovery of endothelial cell permeability. Consequently, BM-MSCs have the potential to prevent radiation-induced microvascular damage and subsequent SG hypofunction. 16 39

Furthermore, it has been proposed that BM-MSCs stimulate radiation-surviving stem/progenitor cells to improve tissue healing by secreting fibroblast growth factor 7 (FGF7) and keratinocyte growth factor (KGF). The proliferation of SG stem cells that had survived radiation treatment was enhanced by the use of KGF after radiotherapy. Saliva flow, endothelial cell count, and acinar cell proliferation were all enhanced in murine SMGs upon adenovirus vector-based transfer of human KGF gene therapy. 21 40 Despite the promising results of the current study, determining the optimal dosage and timing of stem cell administration to achieve maximal regeneration while minimizing side effects may require further investigation. Besides, evaluating functional outcomes following stem cell therapy, including salivary flow rate and quality of saliva production, also needs further research.

## The More Appropriate Conclusion

BM-MSCs have a regeneration potential on induced damaged submandibular SGs in mice; time is an essential factor in the regeneration capacity of BM-MSCs.
